# Explainable Machine‐Learning Model to Classify Culprit Calcified Carotid Plaque in Embolic Stroke of Undetermined Source

**DOI:** 10.1111/jon.70119

**Published:** 2026-01-22

**Authors:** Yu Sakai, Jiehyun Kim, Huy Q. Phi, Andrew C. Hu, Pargol Balali, Konstanze V. Guggenberger, John H. Woo, Daniel Bos, Scott E. Kasner, Brett L. Cucchiara, Luca Saba, Zhi Huang, Daniel Haehn, Jae W. Song

**Affiliations:** ^1^ Department of Radiology University of Pennsylvania Philadelphia Pennsylvania USA; ^2^ Department of Computer Science University of Massachusetts Boston Boston Massachusetts USA; ^3^ Drexel University College of Medicine Philadelphia Pennsylvania USA; ^4^ Perelman School of Medicine University of Pennsylvania Philadelphia Pennsylvania USA; ^5^ Department of Neurology University of Pennsylvania Philadelphia Pennsylvania USA; ^6^ Department of Radiology University of Wuerzburg Wuerzburg Germany; ^7^ Department of Epidemiology Erasmus MC University Medical Center Rotterdam the Netherlands; ^8^ Department of Radiology and Nuclear Medicine Erasmus MC University Medical Center Rotterdam the Netherlands; ^9^ Department of Radiology University of Cagliari Cagliari Italy; ^10^ Department of Pathology and Laboratory Medicine University of Pennsylvania Philadelphia Pennsylvania US

**Keywords:** atherosclerosis, calcification, computed tomography, machine learning, plaque, stroke

## Abstract

**Background and Purpose:**

Embolic stroke of undetermined source (ESUS) may be associated with carotid artery plaques with <50% stenosis. Plaque vulnerability is multifactorial, possibly related to intraplaque hemorrhage (IPH), lipid‐rich necrotic core, perivascular adipose tissue (PVAT), and calcifications. Machine learning (ML)‐based plaque classification is increasingly popular but often limited in clinical interpretability by black‐box nature. We applied an explainable ML approach, using noncalcified plaque components and calcification features with the SHapley Additive exPlanations (SHAP) framework to classify plaques as culprit or nonculprit.

**Methods:**

This was a retrospective, cross‐sectional study. Patients with unilateral anterior circulation ESUS with calcified carotid plaques in neck computed tomography (CT) angiography were analyzed. Calcification‐level features were derived from manual segmentations. Plaque‐level features were assessed by a neuroradiologist and by semi‐automated software. Plaques were classified as culprit if ipsilateral to stroke side. Eight classifiers were benchmarked, and a gradient‐boosted decision tree (CatBoost) was further tuned. SHAP explained model decisions.

**Results:**

Seventy patients yielded 116 calcified plaques (270 calcifications). Model based on five plaque‐ and calcification‐level features achieved ROC‐AUC (receiver operating characteristic area under the curve) 0.79 and precision‐recall‐AUC 0.86, outperforming classification based on plaque thickness ≥3 mm (ROC‐AUC 0.59, *p* = 0.04) and IPH presence (ROC‐AUC 0.51, *p* = 0.003). SHAP identified plaque thickness and PVAT volume as the most influential features with potential thresholds of >2.6 mm and ≥112 mm^3^, respectively.f

**Conclusions:**

ML model trained with noncalcified plaque and calcification features can classify culprit calcified carotid plaque better than conventional criteria. Using clinically interpretable features with SHAP, the model explained its decisions and suggested hypothesis‐generating thresholds.

## 1 Introduction

Embolic stroke of undetermined source (ESUS) affects nearly one in six ischemic stroke patients and carries a recurrence rate of ∼5% per year despite antithrombotic therapy [1, 2]. By definition, ESUS excludes patients with ≥50% stenosis of the artery supplying the infarct territory, yet growing evidence suggests that nonstenotic and mildly stenotic (<50%) carotid plaques can be potential embolic sources in this population. Identifying which of these plaques are truly “vulnerable” remains a major clinical challenge.

Plaque vulnerability is multifactorial with multiple potential imaging features for risk assessment [3]. In the recently introduced carotid Plaque‐RADS classification, wall thickness is a key feature, with a cutoff at ≥3 mm separating low and higher‐risk plaques [4]. Presence of intraplaque hemorrhage (IPH) denotes high‐risk plaque; patients with <50% stenotic plaque with IPH have an elevated risk of recurrent stroke [5]. Plaque‐RADS likewise classifies any plaque with IPH as high‐risk, independent of plaque thickness. Plaque calcification may also be contributory, but its effect appears heterogeneous: microcalcifications and low‐density calcifications have been linked to higher risk, whereas dense macrocalcifications may reflect a potentially stabilizing phenotype [6, 7, 8]. Thus, the size, density, and distribution of individual calcified foci may carry different risk information than aggregate calcification burden. Inflamed adipose tissue surrounding the carotid artery may also indicate vulnerability [9]. These attributes may interact in complex, potentially nonlinear ways that are not fully captured by single‐variable criteria.

Machine learning (ML) offers a means to model such multivariate nonlinear structures [10], and has been applied to stroke imaging [11]. However, prior ML studies of carotid plaque vulnerability have primarily examined patients with ≥50% stenosis rather than those with ESUS [12, 13, 14]. Furthermore, many high‐performing ML models are “black boxes,” limiting clinical adoption; explainable ML with clinically interpretable features is needed [15].

We sought to address this gap by developing and evaluating an explainable ML classifier to distinguish culprit from nonculprit calcified carotid plaques in ESUS with <50% stenosis. Our approach integrates plaque‐level noncalcified volumetric composition with calcification‐level features of individual calcified foci derived from CT angiography (CTA), rather than relying on aggregate calcification burden. The primary objective was to quantify plaque‐level discrimination using receiver operating characteristic area under the curve (ROC‐AUC) of this combined‐plaque‐calcification feature model and use it to compare prediction models using only plaque‐level features and conventional non‐ML‐based criteria (plaque thickness ≥3 mm, IPH presence). The secondary objective was to explain model predictions using SHapley Additive exPlanations (SHAP) and to identify clinically interpretable contributors and potential risk thresholds.

## 2 Methods

The data that support the findings of this study are available from the corresponding author upon reasonable request. This single‐center retrospective cross‐sectional study was deemed exempt by the University of Pennsylvania Institutional Review Board (853355) with waiver of informed consent. Reporting followed Strengthening the Reporting of Observational Studies in Epidemiology (STROBE) [16] and the Checklist for Artificial Intelligence in Medical Imaging (CLAIM) [17] guidelines.

### 2.1 Patient Selection

Consecutive patients with unilateral anterior circulation acute ischemic stroke between October 1, 2015 and April 1, 2017, were screened. Inclusion criteria: age ≥18 years, neck CTA within 10 days of stroke onset, diagnosis of ESUS by vascular neurologists, and confirmation of carotid plaque calcifications on CTA by visual inspection. ESUS was defined per criteria previously reported [18]: infarct greater than 1.5 cm in diameter confirmed on neuroimaging or with vessel occlusion on angiography; absence of myocardial infarction or atrial fibrillation (electrocardiography and minimum of 24 h of telemetry); no cardioembolic source on transthoracic echocardiogram; no ≥50% extra‐ or intracranial stenosis in the infarct territory on vessel imaging; and no other etiology of stroke [1]. Exclusion criteria: patients with multiple possible mechanisms of ischemic stroke, evidence of simultaneous acute ischemic stroke in more than one vascular territory, prior carotid endarterectomy or stenting, or occlusion of either cervical internal carotid artery.

### 2.2 Definition of Culprit Versus Nonculprit Plaque/Calcification

In this ESUS cohort, each patient contributed at least one and at most two plaques: one plaque from the carotid artery ipsilateral to ischemia, and one plaque from the contralateral carotid artery. Cervical carotid plaques and associated calcifications were defined as “culprit” if ipsilateral to the stroke, and “nonculprit” if contralateral. Individual calcifications inherited the label of their parent plaque (e.g., a calcification in the ipsilateral plaque is labeled as culprit).

### 2.3 Imaging Acquisition

Neck CTA was acquired in the axial plane using a fourth‐generation, helical CT scanner (slice thickness 0.625−1.5 mm) after 100 mL of Isovue‐370 intravenous contrast via ≥20‐gauge antecubital catheter.

### 2.4 Image Analysis

All evaluations were blinded to stroke side.

#### 2.4.1 Plaque‐Level Features

A neuroradiologist measured maximal plaque thickness, ulceration, and stenosis according to the North American Symptomatic Carotid Endarterectomy Trial (NASCET). Cases with ≥50% stenosis were excluded.

Each carotid plaque was processed using the commercialized software ElucidVivo (v.2.0, Elucid Bioimaging Inc., Wenham/Boston, MA; http://www.elucidbio.com) to quantify plaque components, as previously described [19]. Briefly, neck CTA was imported, a 4‐cm segment centered on the carotid bifurcation was defined, and the software performed automated lumen and outer wall segmentations, which were visually reviewed and corrected as needed by a board‐certified neuroradiologist. ElucidVivo then classified plaque voxels into tissue classes—lipid‐rich necrotic core (LRNC), IPH, calcification, matrix (defined as plaque tissue not belonging to other components), and perivascular adipose tissue (PVAT)—and reported volumetric measures for each class [20]. A detailed description and validation of the software workflow have been published elsewhere [21].

#### 2.4.2 Calcification‐Level Features

Both carotid arteries were screened across a 4‐cm segment centered on the bifurcation by two independent trained evaluators with consensus by radiologists. Arteries without visible calcified plaque were excluded. A single plaque could contain multiple discrete (noncontiguous) calcification foci, each with distinct size, shape, and attenuation. In contrast to plaque‐level calcification metrics, which summarize the aggregate calcification burden of each plaque (e.g., total volume and mean attenuation), calcification‐level features characterize the heterogeneity of individual foci within the same plaque. This per‐calcification representation allows capture of potentially high‐risk patterns such as low‐density or relatively large individual calcifications that might be obscured when only plaque‐level averages are used. Therefore, each discrete calcification was segmented in 3D Slicer (v.5.6, The Slicer Community, Boston, MA, https://www.slicer.org) independently by two trained evaluators, with consensus review by a neuroradiologist. Segmentation maps were used to derive calcification morphology (volume, maximum diameter, surface area, attenuation). Attenuation values are reported in Hounsfield Units (HU). Each calcification was also manually scored by a trained evaluator for its location relative to the vessel wall (adventitial, intimal, or transmural) and arc angle, the maximum circumferential extent of the calcification around the arterial lumen to derive “spotty calcification” [6, 22] and “rim‐sign” following prior definitions [7].

### 2.5 ML Methods

All analyses were performed in Python (v3.10.12, Python Software Foundation, Wilmington, DE, USA; https://www.python.org) using scikit‐learn, xgboost, catboost, and lightgbm packages.

#### 2.5.1 Input Data

The dataset comprised plaque‐level features characterizing each carotid artery plaque, along with nested calcification‐level features describing individual calcified components within each plaque. Baseline feature distributions were tested for normality (Shapiro−Wilk) and compared between ipsilateral and contralateral sides (Wilcoxon rank‐sum or *t*‐test).

#### 2.5.2 Training and Test Set Split

Patients admitted in 2015–2016 comprised the training set; patients admitted in 2017 comprised the test set, simulating prospective validation.

#### 2.5.3 Feature Engineering and Selection

On the training set, feature collinearity was assessed with variance inflation factor (VIF > 5) and Pearson's correlation; feature pairs with |*r*| > 0.3 were filtered, reflecting a conservative threshold for this modest sample. Feature importance ranking was then performed with a shallow decision tree (max depth = 3), and the top five features were retained. To minimize model instability from outliers in a modest sample, continuous variables were discretized into deciles; cut points were defined using the training set and applied unchanged to the test set to prevent data leakage.

#### 2.5.4 Algorithm Selection

First, baseline performances of eight common classifiers (logistic regression, SVM [support vector machine], decision tree, random forest, naïve Bayes, XGBoost, CatBoost, LightGBM) [23, 24, 25, 26] were compared without any hyperparameter tuning using 10‐fold group cross‐validation, grouping by plaque ID so that calcifications from the same plaque did not appear in both training and validation folds. CatBoost, the top‐performing classifier, was then tuned and used for subsequent analyses.

#### 2.5.5 Model Training and Validation

The CatBoost model was tuned by grid search (learning rate 0.001−0.1, depth 3–10, iterations 50–500) using 10‐fold group cross‐validation, grouped by plaque ID so that calcifications from the same plaque did not cross folds.

#### 2.5.6 Model Comparisons

Four models were compared:

Model 1 (Combined): A CatBoost classifier trained using the five calcification‐ and plaque‐level inputs. Because the lowest unit of analysis was individual calcification (a single plaque can contain multiple calcifications), the classifier first produced predictions at the calcification level. For plaques containing multiple calcifications, we then averaged the predicted probabilities across all calcifications within that plaque to obtain a single plaque‐level probability; for plaques with a single calcification, the plaque‐level probability equaled the calcification‐level probability.
Model 2 (Plaque‐only): CatBoost classifier trained using only plaque‐level features.
Model 3 (Thickness criteria): Culprit plaque if plaque thickness ≥3 mm.
Model 4 (IPH criteria): Culprit plaque if IPH volume >0.

#### 2.5.7 Performance Metrics and Comparison

Discrimination was quantified with the area under the ROC curve (ROC‐AUC) and the area under the precision–recall curve (PR‐AUC). Paired comparisons of ROC‐AUC between the Combined model (Model 1) and comparators (Models 2–4) were performed using the DeLong test for correlated ROC curves, reporting ΔAUC, 95% CIs, and two‐sided *p*‐values. For PR‐AUC, 95% CIs were obtained via nonparametric bootstrap (10,000 resamples) [27]. Accuracy, precision, recall, and F1 score were calculated. Calibration (plaque‐level), defined as the agreement between predicted probabilities and the observed frequency of culprit plaques, was assessed using the Brier score, expected calibration error (ECE; 10 quantile bins), and calibration slope from logistic regression of observed outcomes on predicted log‐odds. We refer to the raw CatBoost predicted probabilities, obtained directly from the classifier before any scaling or recalibration, as “uncalibrated probabilities.” Post‐hoc calibration used cross‐validated out‐of‐fold training predictions to fit isotonic regression (primary) and Platt scaling (secondary), thereby avoiding information leakage; each calibrator was then applied once to the held‐out test set. Calibrated probabilities were treated as exploratory and not used for clinical risk stratification.

#### 2.5.8 Interpretability and Error Analysis

SHAP values were used to quantify feature contributions and visualize directionality [28]. A confusion matrix was generated. SHAP waterfall plots were examined to analyze misclassifications.

## 3 Results

### 3.1 Cohort

**FIGURE 1:**
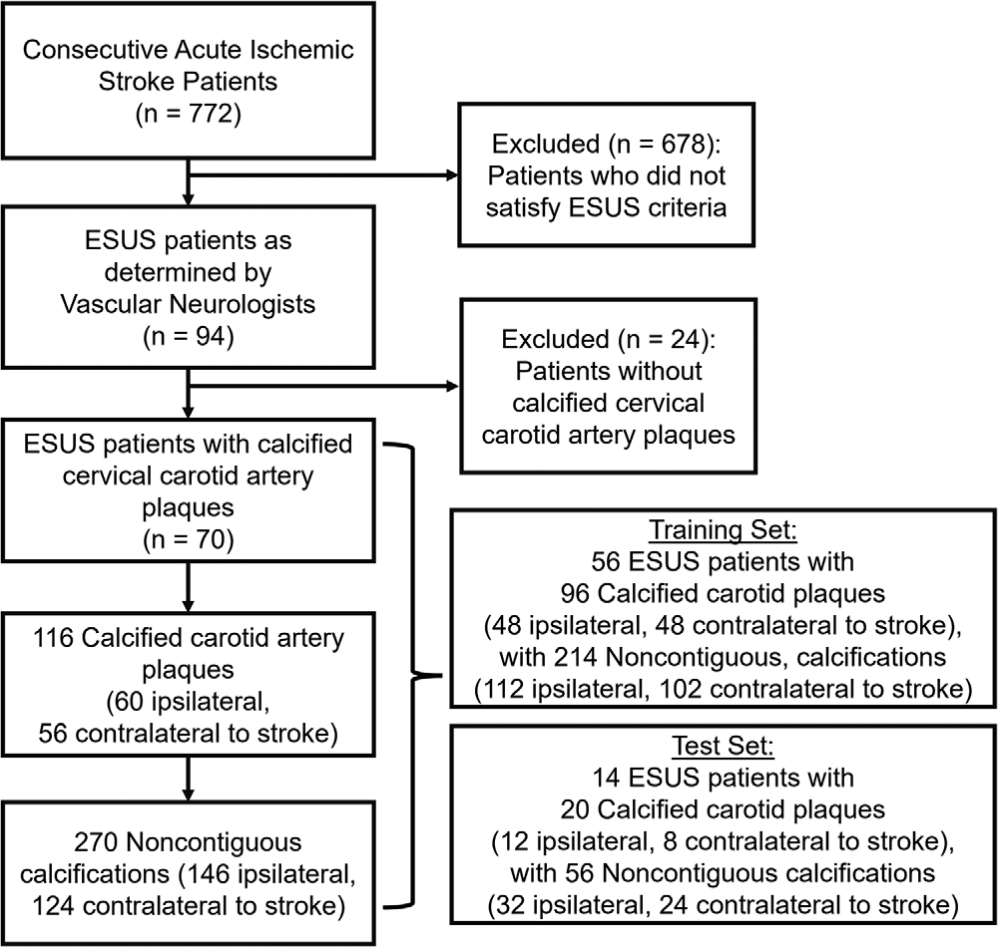
Patient flow diagram. Flow diagram shows 70 patients with embolic stroke of undetermined source (ESUS), yielding 116 calcified plaques and 270 calcifications. Abbreviation: *n*, number of patients.

From the 772 acute ischemic stroke patients screened, 94 met ESUS criteria. Twenty‐four were excluded for absence of carotid calcification, yielding 70 patients (mean age 68 ± 11 years, 50% women) (Figure 1 and Table 1).

**TABLE 1:** Demographic data.

Total dataset *n* = 70				
Characteristic	Mean (standard deviation)	*n* (%)	Training set *n* = 56	Test set *n* = 14
Age	68 (11)		67 (10)	71 (14)
Sex				
Female		35 (50%)	26 (46%)	9 (64%)
Diabetes		22 (31%)	18 (32%)	4 (29%)
Hypertension		53 (76%)	44 (79%)	9 (64%)
Coronary artery disease		19 (27%)	16 (29%)	3 (21%)
Dyslipidemia		28 (40%)	18 (32%)	10 (71%)
Smoker		14 (20%)	12 (21%)	2 (14%)

Abbreviation: *n*, number of patients.

### 3.2 Training and Test Split

The training set included 56 patients with 96 plaques and 214 calcifications; the test set included 14 patients with 20 plaques and 56 calcifications.

### 3.3 Imaging Features

One hundred and sixteen calcified carotid plaques (60 ipsilateral, 56 contralateral) were identified, comprising 270 discrete calcifications (146 ipsilateral, 124 contralateral, median of 2 calcifications per plaque with interquartile range 1–3). Seventeen plaque‐level and 12 calcification‐level features were initially extracted (Table 2).

**TABLE 2:** Initial derived features.

Feature	Definition	Ipsilateral median [Q1–Q3]	Contralateral median [Q1–Q3]	*p*‐value
**Calcification‐level features**				
Arc angle	Angular coverage of calcification; 1 = 0−90°, 2 = 90−180°, 3 = 180−270°, 4 = 270−360°.	1.0 [1.0–2.0]	1.0 [1.0–2.0]	0.99
Location	Position in vessel wall; 1 = intimal, 2 = adventitial, 3 = transmural.	3.0 [3.0–3.0]	3.0 [2.0–3.0]	0.33
Spotty calcification	Arc <90° and thickness <3 mm	0 [0–1]	0 [0–1]	0.19
Volume (mm^3^)	Segmentation volume	25.0 [10.2–56.7]	17.9 [7.8–38.7]	0.06
Diameter (mm)	Feret diameter	5.39 [3.32–8.03]	4.70 [2.74–7.45]	0.10
Surface area (mm^2^)	Segmentation surface	43.5 [21.1–81.1]	36.6 [17.3–62.4]	0.07
Minimum attenuation (HU)	Lowest HU within calcification	210.5 [150.8–262.8]	194.0 [152.8–239.5]	0.18
Maximum attenuation (HU)	Highest HU within calcification	918.5 [614.0–1400.5]	733.5 [529.8–1204.5]	0.06
Mean attenuation (HU)	Mean HU within calcification	513.2 [415.2–693.4]	468.4 [326.4–634.3]	0.05
Volume/min attenuation	Volume/minimum HU	0.118 [0.046–0.391]	0.098 [0.035–0.248]	0.13
Volume/max attenuation	Volume/maximum HU	0.027 [0.017–0.045]	0.022 [0.014–0.043]	0.17
Volume/mean attenuation	Volume/mean HU	0.050 [0.027–0.098]	0.040 [0.022–0.078]	0.20
**Plaque‐level features**				
Thickness (mm)	Maximal thickness	3.1 [2.8–3.8]	2.8 [2.4–3.8]	0.05
Ulceration	≥2 mm indentation of lumen	0 [0–0]	0[0–0]	0.14
Plaque matrix (mm^3^)	Fibrous tissue	829.5 [654.9–966.5]	795.1 [594.2–976.7]	0.40
IPH (mm^3^)	Intraplaque hemorrhage	8.67 [2.31–13.75]	5.33 [2.53–12.71]	0.46
LRNC (mm^3^)	Lipid‐rich necrotic core	71.3 [27.5–112.9]	71.5 [26.5–139.1]	0.80
LRNC—IPH volume	LRNC minus IPH	57.1 [17.3–110.8]	55.9 [21.5–130.9]	0.60
IPH/LRNC	Ratio IPH/LRNC	0.104 [0.032–0.367]	0.101 [0.029–0.308]	0.55
PVAT (mm^3^)	Perivascular adipose tissue	121.5 [34.6–191.3]	105.0 [51.0–218.1]	0.88
Total plaque (mm^3^)	Total plaque volume	966.0 [777.1–1258.1]	920.6 [730.2–1207.6]	0.13
IPH/total volume	Ratio IPH/total	0.006 [0.002–0.010]	0.005 [0.002–0.009]	0.57
LRNC/total volume	Ratio LRNC/total	0.051 [0.020–0.090]	0.063 [0.030–0.095]	0.34
PVAT/total volume	Ratio PVAT/total	0.088 [0.029–0.149]	0.072 [0.033–0.167]	0.83
Total calc vol (mm^3^)	Total calcifications	133.6 [57.2–261.2]	76.4 [39.0–179.7]	0.01
Mean calc attenuation (HU)	Average HU of calcifications	529.6 [445.5–621.8]	467.1 [396.9–633.6]	0.01
IPH+LRNC (mm^3^)	Sum of IPH, LRNC	82.4 [37.3–141.8]	80.4 [39.2–146.0]	0.76
Total calc vol/mean attenuation	Ratio calcification volume/mean HU	0.268 [0.116–0.449]	0.168 [0.082–0.333]	0.02
Rim sign	Presence of adventitial calc >90° arc + ≥2 mm plaque	0 [0–0]	0 [0–0]	0.54

*Note*: List of initially derived features, from which five were selected for the final Combined model.

Abbreviations: Calc, calcification; HU, Hounsfield units; IPH, intraplaque hemorrhage; LRNC, lipid‐rich necrotic core; PVAT, perivascular adipose tissue; Q, quartile.

### 3.4 Feature Selection

**FIGURE 2:**
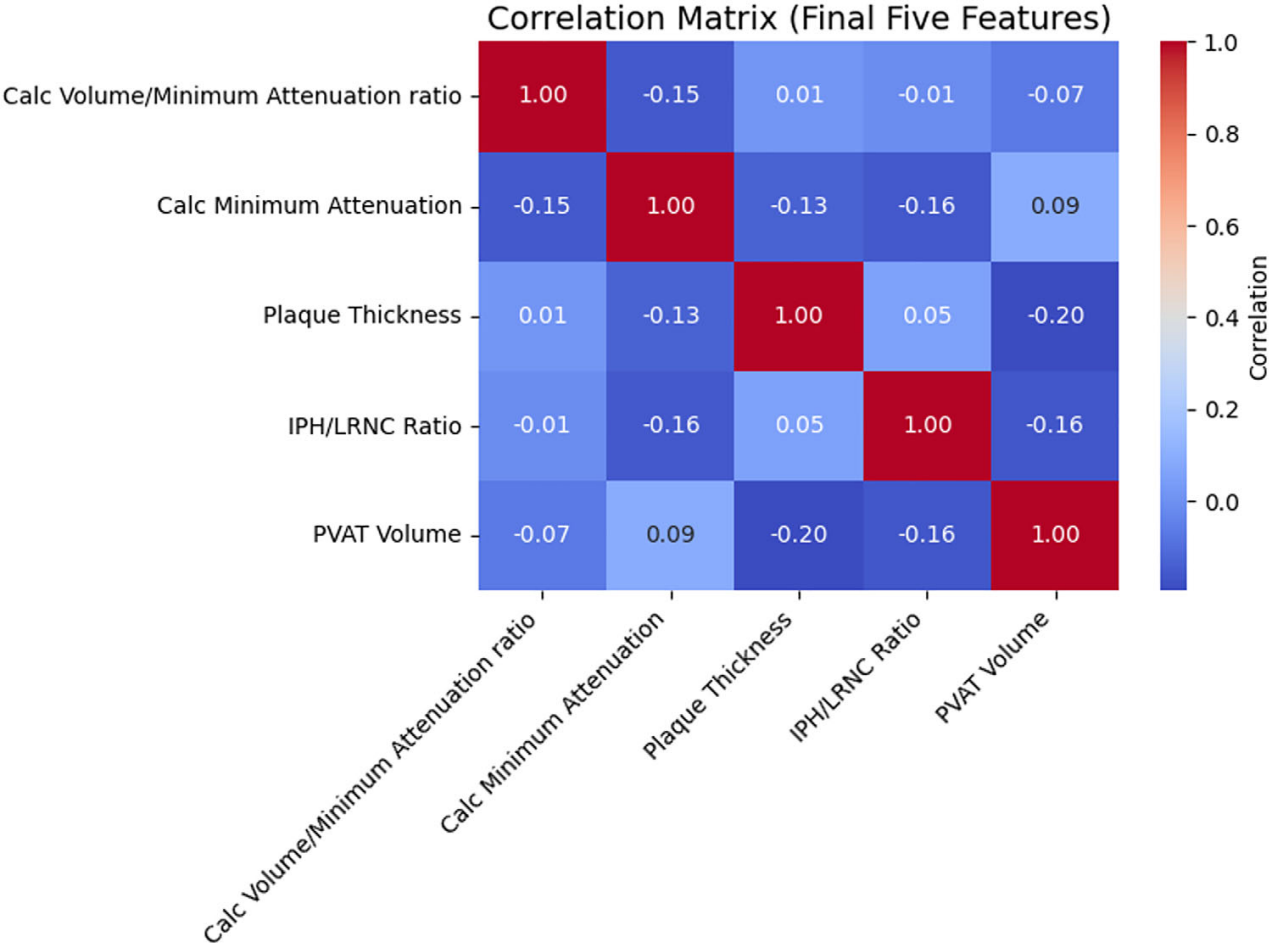
Pearson correlation matrix for Combined model features. Heatmap shows pairwise Pearson correlation coefficients (*r*) among the five features (calcification volume/minimum attenuation ratio, calcification minimum attenuation, plaque thickness, intraplaque hemorrhage [IPH]/lipid‐rich necrotic core [LRNC] ratio, perivascular adipose tissue [PVAT] volume) retained in the Combined model. All retained features demonstrated low collinearity (|*r*| < 0.3). Abbreviation: Calc, calcification.

**FIGURE 3:**
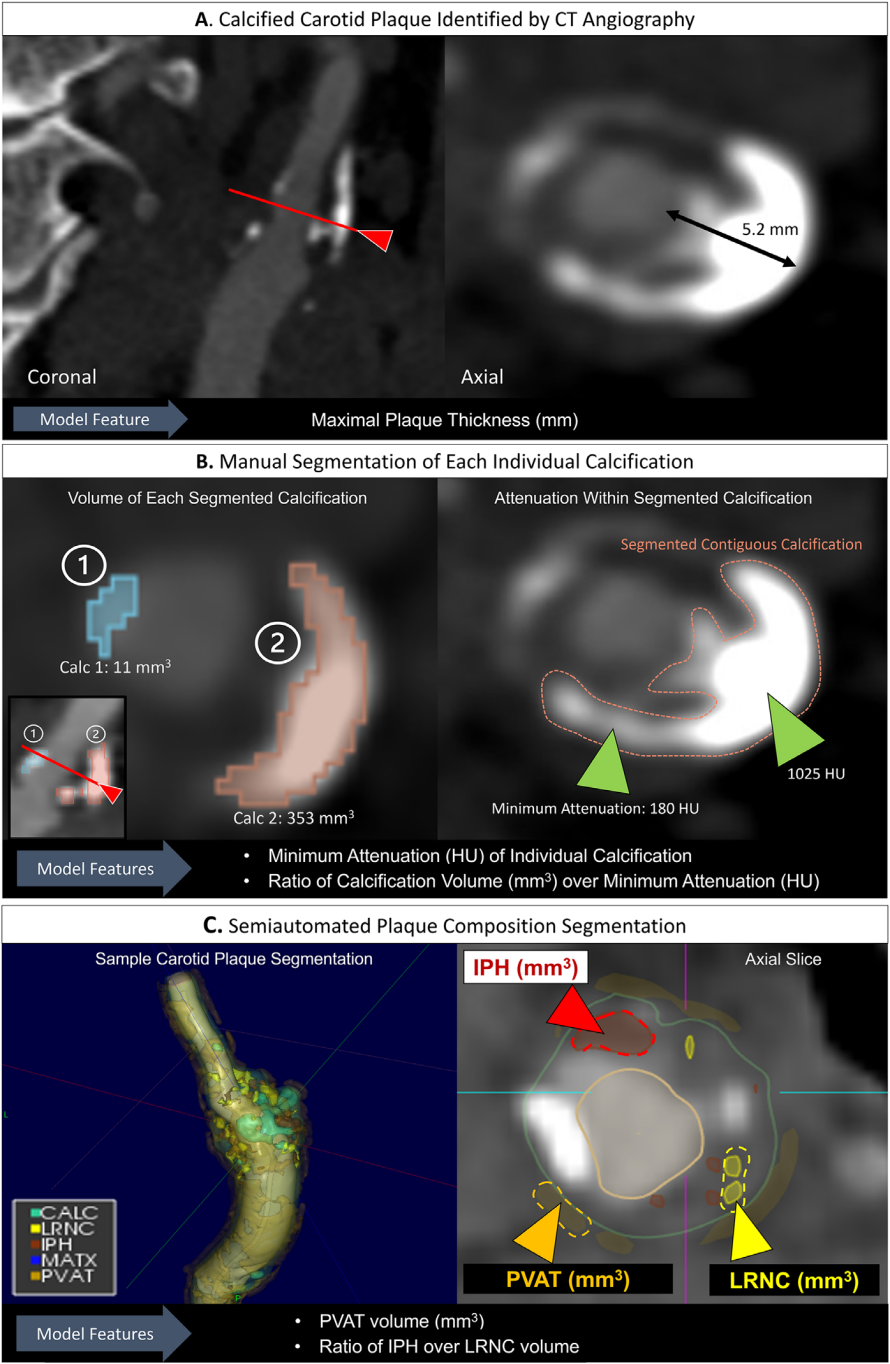
Schematic of imaging features derivation for Combined model. (A) CT angiography images of carotid artery in coronal (left) and axial (right) views; cross‐sectional inset provided (red line with arrow). Maximal plaque thickness was measured by neuroradiologist from luminal outer wall to the plaque outer wall (black double‐arrow) and was used as feature for the final Combined model. (B) Sample segmentation of individual calcifications in 3D slicer. Left image shows axial slice of a plaque with two calcifications (Calc 1 and Calc 2, blue and orange segmentations, respectively). Subpanel (left bottom corner) shows coronal view of plaque with cross‐sectional inset (red line with arrow) for anatomical reference. Each calcification has different volume; 11 mm^3^ for Calc 1 and 353 mm^3^ for Calc 2. Right image demonstrates variable internal attenuation within a contiguous calcification (orange dashed borders): area of minimum attenuation of 180 HU and area with over 1000 HU (green arrowheads). Calcification‐level features used for final Combined model: 1. minimum attenuation (HU) of individual calcification and 2. ratio of calcification volume (mm^3^) over minimum attenuation (HU). (C) Sample semiautomated segmentation of plaque‐level compositions using Elucid Bioimaging software. Left image shows 3D segmentation of the entire carotid plaque by Elucid software, into five components: calcification (CALC, green), lipid‐rich necrotic core (LRNC, yellow), intraplaque hemorrhage (IPH, brown), matrix (MATX, blue), and perivascular adipose tissue (PVAT, orange). Right image shows example axial view of the segmentation with intraplaque hemorrhage (IPH) (red dashed‐line outline, red arrowhead), lipid‐rich necrotic core (LRNC) (yellow dashed‐line outline, yellow arrowhead), and perivascular adipose tissue (PVAT) (orange dashed‐line outline, orange arrowhead) highlighted. Plaque‐level features used for final Combined model: 1. PVAT volume (mm^3^), 2. ratio of IPH over LRNC volume. Abbreviation: Calc, calcification;IPH, Intraplaque hemorrhage; HU, Hounsfield Unit; LRNC, Lipid rich necrotic core; MATX; Matrix, PVAT, Perivascular adipose tissue.

Five independent predictors were retained after collinearity filtering and decision‐tree ranking: ratio of IPH over LRNC volume, maximal plaque thickness, PVAT volume, calcification minimum attenuation (calcification‐level metric defined as minimum attenuation within an individual calcification), and ratio of calcification volume over minimum attenuation (calcification‐level metric defined as the ratio of an individual calcification's volume over its minimum attenuation) (Figure 2). Representative schematic of the derivation process for these imaging features is shown in Figure 3.

### 3.5 Model Development

**FIGURE 4:**
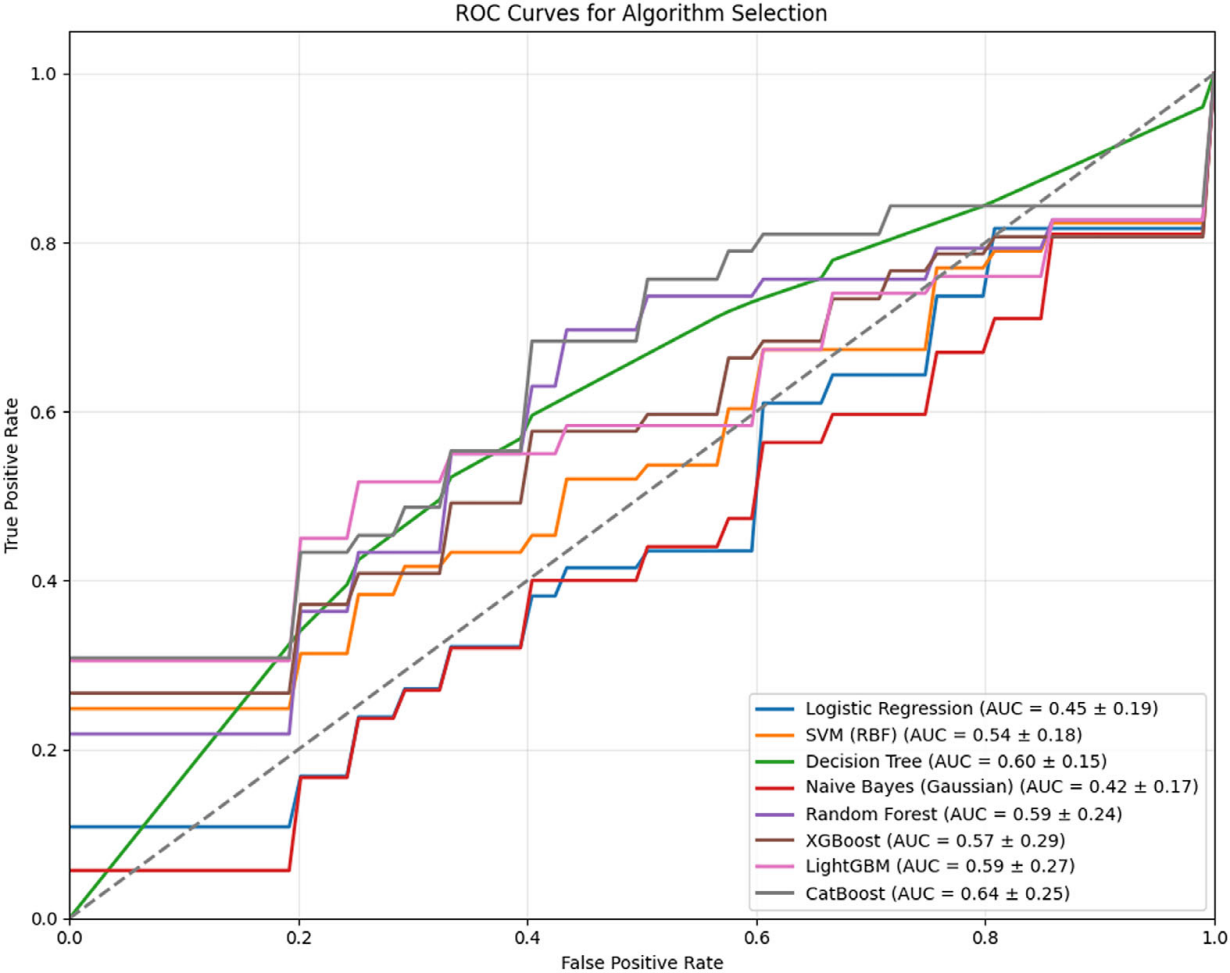
Receiver operating characteristic curves of eight benchmark ML models. ROC curves comparing eight classifiers for algorithm selection; CatBoost had the highest mean cross‐validation ROC‐AUC (0.64). All the data represent mean ± standard deviation unless otherwise indicated. Abbreviations: ML, machine learning; RBF, radial basis function kernel; ROC‐AUC, receiver operating characteristic curve area under the curve; SVM, support vector machine.

Among eight classifiers benchmarked, CatBoost performed best (cross‐validation ROC‐AUC 0.64) (Figure 4). Tuned hyperparameters: learning rate 0.001, depth 9, iterations 50.

### 3.6 Model Performance and Comparison

**FIGURE 5:**
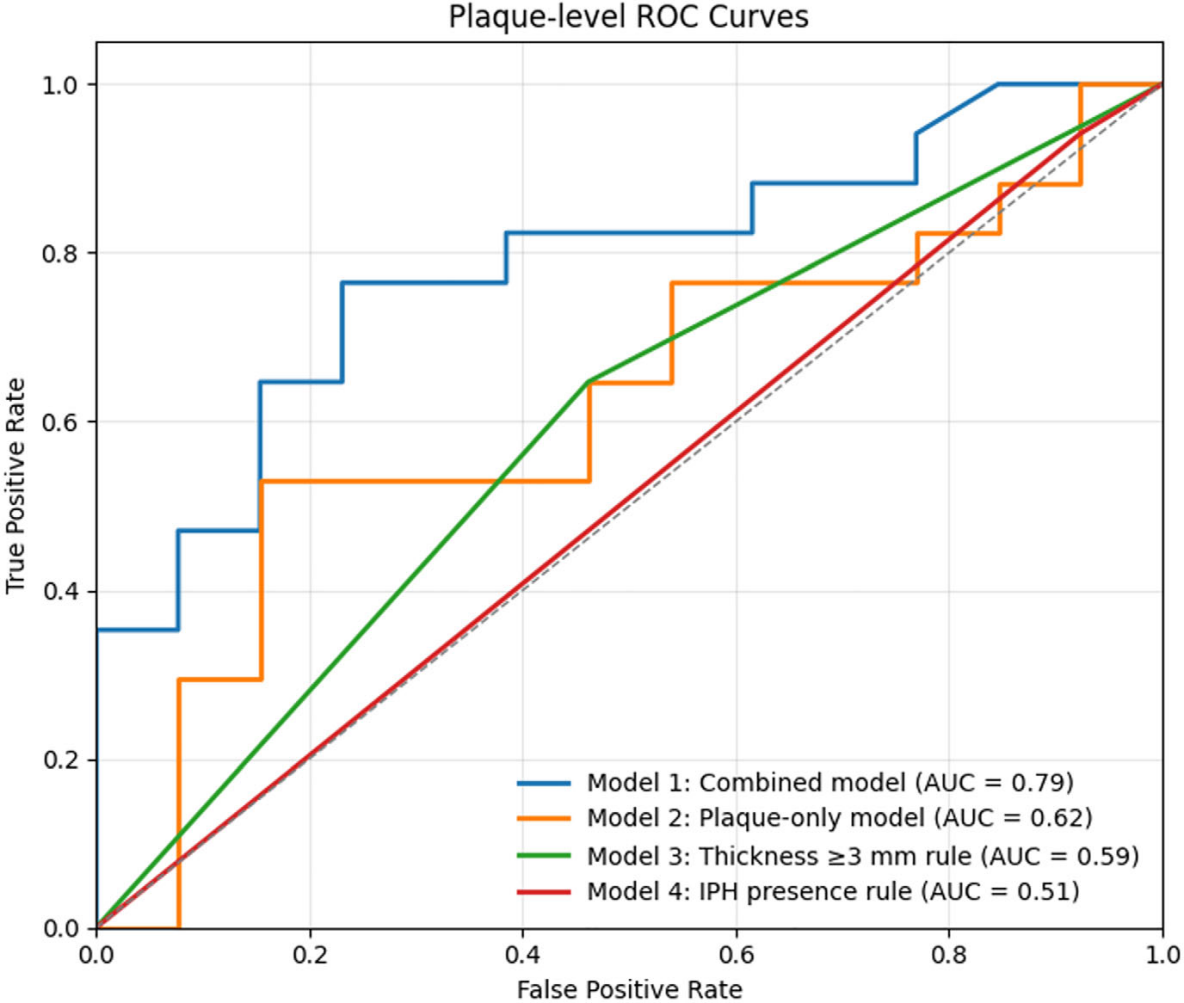
Receiver operating characteristic curve of models in culprit plaque classification. ROC curves for culprit plaque classification with best performance by Combined model (test ROC‐AUC 0.79). Abbreviations: IPH, intraplaque hemorrhage; ROC‐AUC, receiver operating characteristic curve area under the curve.

After tuning, Model 1 (Combined model) achieved a mean cross‐validation ROC‐AUC 0.73 and test set plaque‐level ROC‐AUC 0.79 (95% CI 0.55–0.97) (Figure 5 and Table 3).

**TABLE 3:** Combined model performance on test set.

Metric	Plaque‐level performance	Calc‐plaque pair level performance
ROC‐AUC	0.79 (95% CI: 0.55–0.97)	0.79 (95% CI: 0.66–0.91)
PR‐AUC	0.86 (95% CI: 0.66–0.99)	0.85 (95% CI 0.73–0.94)
Accuracy	0.70 (95% CI: 0.50–0.90)	0.73 (95% CI: 0.61–0.84)
Precision	0.80 (95% CI: 0.50–1.00)	0.79 (95% CI: 0.65–0.92)
Recall	0.67 (95% CI: 0.38–0.92)	0.76 (95% CI: 0.61–0.90)
F1‐Score	0.72 (95% CI: 0.46–0.91)	0.78 (95% CI: 0.65–0.87)

Abbreviations: Calc, calcification; CI, confidence interval; PR‐AUC, precision recall area under the curve; ROC‐AUC, receiver operating characteristic curve area under the curve.

Comparators achieved ROC‐AUC 0.62 (Model 2; trained using only plaque‐level features: thickness, PVAT, IPH/LRNC), 0.59 (Model 3; thickness ≥3 mm), and 0.51 (Model 4; IPH > 0).

DeLong comparisons showed Model 1 outperformed Model 3 (ΔAUC 0.19, 95% CI 0.01−0.38; *p* = 0.04) and Model 4 (ΔAUC 0.28, 95% CI 0.10−0.45; *p* = 0.003). Compared to Model 2, there was a trend toward improvement without reaching significance (ΔAUC 0.17, 95% CI −0.04 to 0.37; *p* = 0.11). The uncalibrated probabilities were overconfident, meaning that plaques with high predicted risk had a lower observed frequency of culprit plaques than the probabilities suggested (Brier 0.25; ECE 0.30; slope 0.05); isotonic calibration improved agreement between predicted and observed risk (Brier 0.22; ECE 0.14; slope 0.92) with only a small, nonsignificant change in ROC‐AUC (0.79→0.73; Δ −0.06, 95% CI −0.17 to 0.05). Platt scaling did not meaningfully affect calibration or ROC‐AUC.

Precision‐recall AUC analysis showed similar observations. At the plaque level, the uncalibrated model achieved PR‐AUC 0.86 (95% CI 0.66–0.99; prevalence 0.60). There was a modest decrease in PR‐AUC after isotonic calibration (0.86 to 0.75; ΔPR‐AUC −0.11, 95% CI −0.23 to −0.01), consistent with slightly reduced discrimination but more realistic probability estimates, whereas Platt scaling had no effect (ΔPR‐AUC 0.00).

### 3.7 Interpretability and Failure Analysis

**FIGURE 6:**
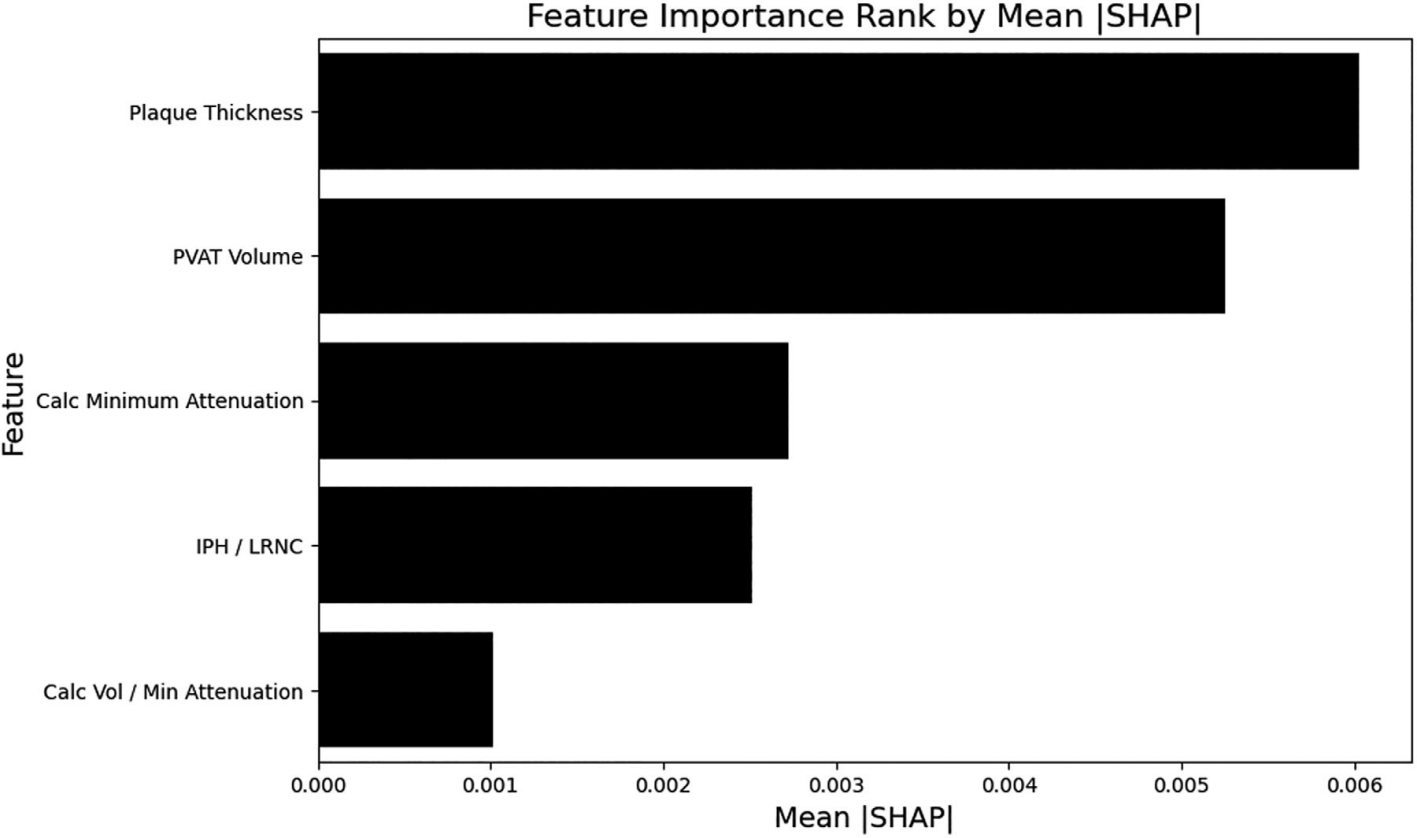
Feature importance ranking of Combined model. Five features used for the Combined model were ranked by mean absolute SHapley Additive exPlanations score, with plaque thickness and perivascular adipose tissue volume being the two most influential. Abbreviations: Calc, calcification; IPH, intraplaque hemorrhage; LRNC, lipid‐rich necrotic core; PVAT, perivascular adipose tissue; SHAP, SHapley Additive exPlanations.

SHAP analysis identified plaque thickness and PVAT volume as the two most influential predictors for our model (Figure 6).

**FIGURE 7:**
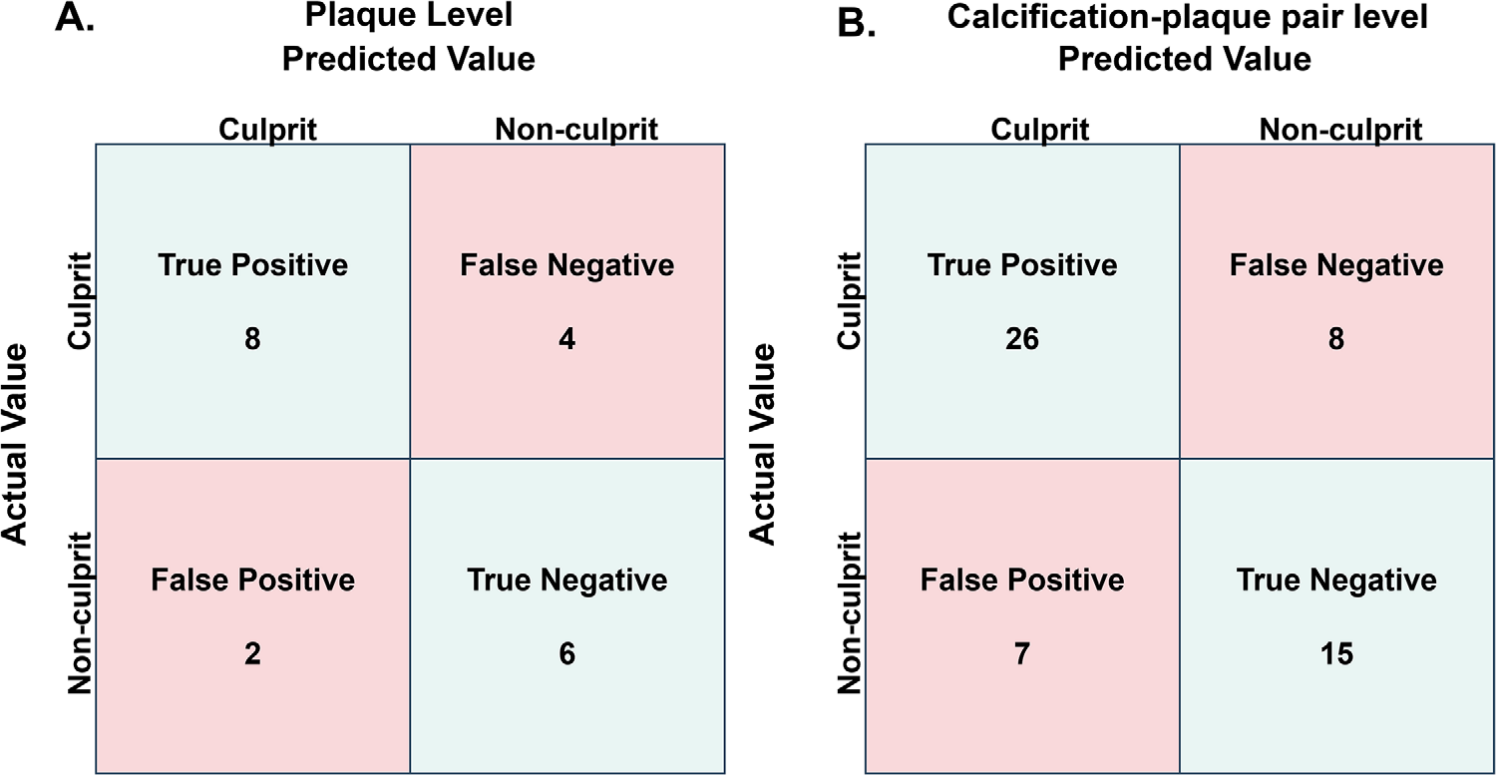
Confusion matrix of Combined model predictions. Confusion matrices for Combined model. (A) Plaque‐level and (B) calcification‐plaque pair level predictions.

The model was more specific than sensitive at plaque‐level prediction, with a confusion matrix showing eight true positives, six true negatives, two false positives, and four false negatives (Figure 7).

**FIGURE 8:**
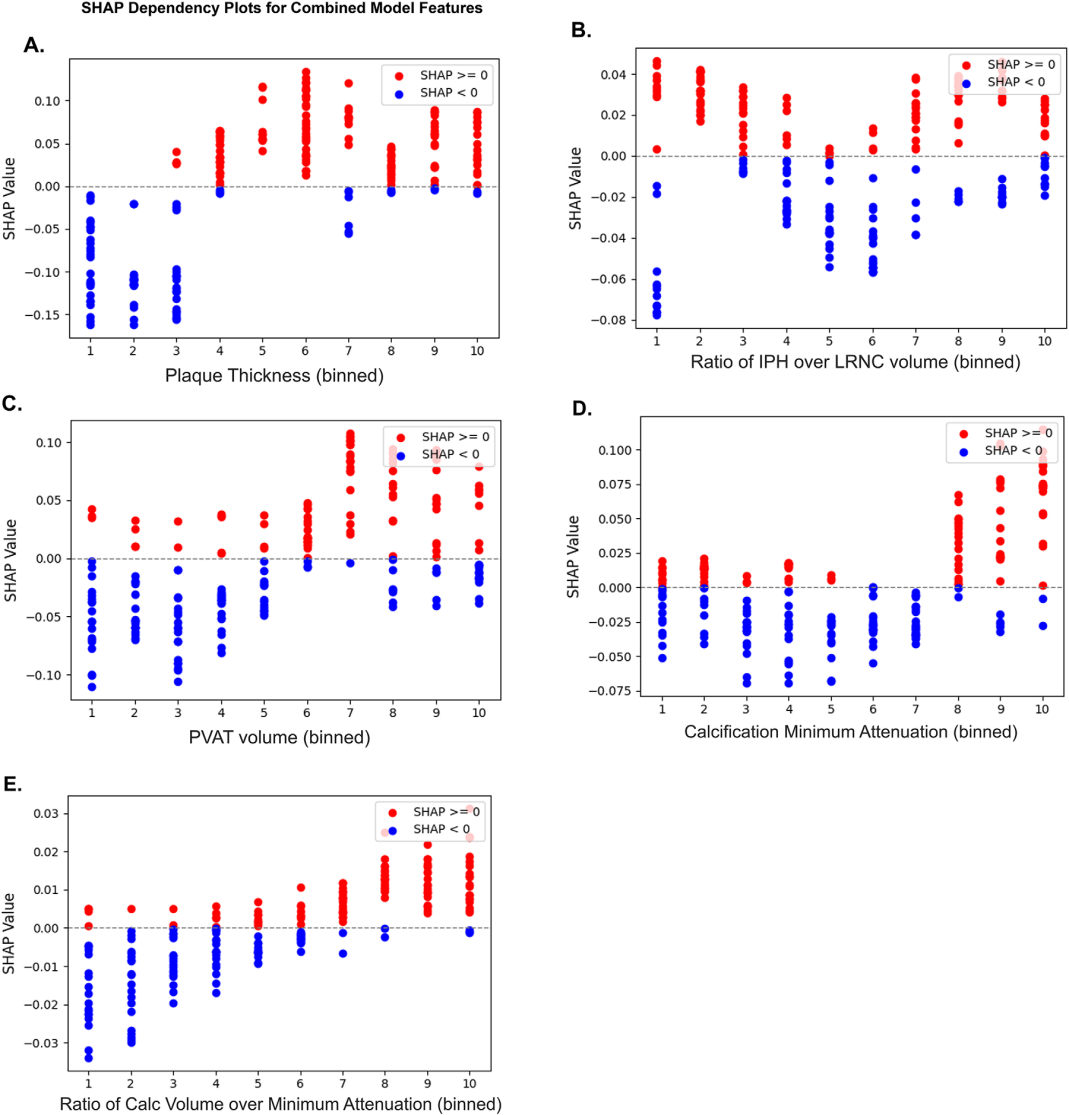
SHAP dependency plots for Combined model features. SHapley Additive exPlanations (SHAP) dependency plots for Combined model features, illustrating the influence of each feature on classification toward culprit (positive, in red color) versus nonculprit (negative, in blue color). All features were discretized into deciles to enhance model stability and reduce the impact of outliers. (A) Plaque thickness, (B) ratio of intraplaque hemorrhage (IPH) over lipid‐rich necrotic core (LRNC) volume, (C) perivascular adipose tissue (PVAT) volume, (D) calcification minimum attenuation, and (E) ratio of calcification volume over minimum attenuation. Approximate thresholds were observed at the fourth decile for plaque thickness, sixth decile for PVAT volume, eighth decile for calcification minimum attenuation, and seventh decile for calcification volume‐to‐minimum attenuation ratio. For IPH/LRNC ratio, a bimodal effect was observed. Abbreviation: Calc, calcification.

SHAP dependency plots revealed exploratory thresholds in feature values associated with shifts in model prediction (Figure 8). Using decile ranges as reference (Table 4), plaque thickness ≥2.6 mm, PVAT volume ≥112 mm^3^, calcification minimum attenuation ≥240 HU, and calcification volume‐to‐minimum attenuation ratio ≥0.15 were associated with increased model contribution toward culprit classification. For the ratio of IPH over LRNC volume, a bimodal pattern was observed, with both very low (<0.02) and higher values (>0.15) contributing positively. These thresholds are exploratory, intended for hypothesis generation, and reflect model behavior within our dataset, rather than definitive clinical cutoffs.

**TABLE 4:** Decile ranges for combined model features.

Decile	IPH/LRNC volume (unitless)	Calcification minimum attenuation (HU)	Plaque thickness (mm)	PVAT volume (mm^3^)	Calcification volume/minimum attenuation (mm^3^/HU)
1	0.00–0.01	−5.00 to 103.30	0.00–2.10	0.00–10.35	−51.76 to 0.02
2	0.01–0.02	103.30–141.40	2.10–2.30	10.35–32.44	0.02–0.03
3	0.02–0.04	141.40–159.70	2.30–2.60	32.44–59.45	0.03–0.05
4	0.04–0.07	159.70–180.00	2.60–2.90	59.45–78.74	0.05–0.07
5	0.07–0.10	180.00–196.00	2.90–3.00	78.74–112.21	0.07–0.10
6	0.10–0.15	196.00–222.20	3.00–3.30	112.21–149.14	0.10–0.15
7	0.15–0.30	222.20–240.10	3.30–3.70	149.14–168.44	0.15–0.21
8	0.30–0.39	240.10–266.40	3.70–4.10	168.44–236.25	0.21–0.41
9	0.39–0.59	266.40–318.70	4.10–4.70	236.25–313.28	0.41–0.83
10	0.59–40.29	318.70–429.00	4.70–6.20	313.28–491.36	0.83–322.95

*Note*: Selected features were discretized into deciles based on training set to reduce instability from outliers during training; multiple variables showed outliers concentrated in the first and/or 10th deciles.

Abbreviations: HU, Hounsfield units; IPH, intraplaque hemorrhage; LRNC, lipid‐rich necrotic core; PVAT, perivascular adipose tissue.

**FIGURE 9:**
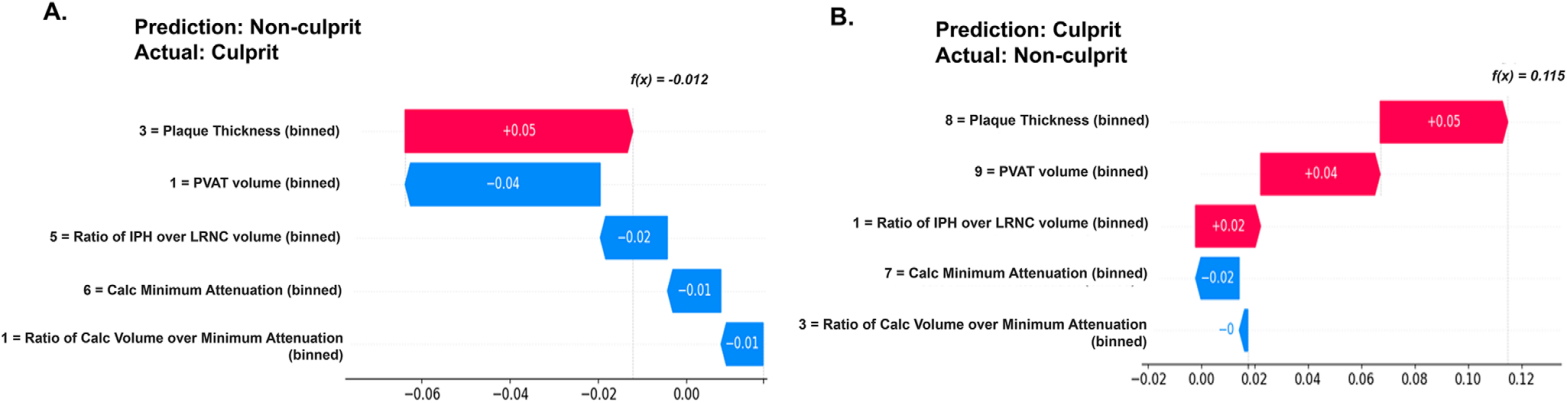
Example SHAP waterfall plots for misclassified cases. Examples of SHapley Additive exPlanations (SHAP) waterfall plots to visualize misclassifications. (A) False negative example, in which plaque thickness favored positive direction (culprit, in red), but negative direction of all other features resulted in incorrect prediction as nonculprit. (B) False positive example, in which calcification features favored negative direction (nonculprit, in blue), but positive direction of all other features resulted in incorrect prediction as culprit. Abbreviations: Calc, calcification; IPH, intraplaque hemorrhage; LRNC, lipid‐rich necrotic core; PVAT, perivascular adipose tissue.

SHAP waterfall plots allowed interpretation of the model's decision‐making process in misclassified cases (Figure 9). In a false‐negative example, high plaque thickness favored the culprit, but the negative direction of all other features resulted in an incorrect prediction as nonculprit. In a false‐positive example, calcification features favored the nonculprit, but the positive direction of all other features resulted in an incorrect prediction as the culprit.

## 4 Discussion

In ESUS patients with <50% carotid stenosis, an explainable CatBoost classifier integrating plaque‐level composition (IPH, LRNC, PVAT, thickness) with calcification‐level metrics (volume and attenuation) achieved strong discrimination of culprit versus nonculprit lesions (ROC‐AUC 0.79). This Combined model outperformed conventional classification based on plaque thickness ≥3 mm and presence of IPH.

Most prior ML studies of carotid vulnerability have focused on ≥50%–70% stenosis and/or radiomic features that are difficult to interpret clinically [12, 13, 14, 29, 30]. The need for interpretable ML is increasingly recognized [15]. Our findings extend this literature by (1) targeting nonstenotic plaques relevant to ESUS, and (2) building a simple model based on five clinically interpretable features with improved explainability via the SHAP framework.

Findings were consistent with prior observations. Maximal plaque thickness, a surrogate for overall plaque burden and positive remodeling [18, 31], emerged as the dominant predictor, with a potential threshold point for plaque thickness (>2.6 mm) aligning with literature and recent Plaque‐RADS linking ≥3 mm thickness as a marker for culprit plaques [4, 18, 31]. IPH is a well‐established vulnerability marker, reflecting the accumulation of blood components within the atheromatous plaque, most often due to leakage from immature, fragile neovessels originating from the vasa vasorum [3, 32, 33]. LRNC refers to the lipid‐dense central region of the plaque, and the ratio of IPH over LRNC volume—reflecting the relative burden of hemorrhagic component to necrotic core—is increasingly recognized as a specific biomarker of plaque vulnerability [13, 32]. PVAT, which can contribute to atherosclerosis through paracrine secretion of proinflammatory adipokines [34], was identified by SHAP as a key contributor in our model, resonating with increasing recognition of its potential role in ESUS [9, 35]. SHAP allowed identification of a potential hypothesis‐generating threshold of PVAT ≥112 mm^3^. In our cohort, plaque thickness (ROC‐AUC 0.59) was superior to IPH presence (ROC‐AUC 0.51) for identifying vulnerable plaques. Adding plaque composition (IPH/LRNC ratio and PVAT volume) and calcification metrics modestly improved performance (0.62, 0.79, respectively). Although these stepwise gains were modest and not statistically significant on pairwise comparison, the final five‐feature Combined model significantly outperformed either plaque thickness criteria or IPH presence alone. The finding that calcification metrics provided incremental but not statistically significant predictive value underscores a nuanced and still uncertain role of calcification attenuation and volume in plaque stability. The relationship of calcification density, volume, and plaque stability remains debated [8, 36, 37, 38, 39]; our findings suggest their interaction may be relevant in ESUS. As a post‐hoc analysis, we tested a model that used aggregated plaque‐level calcification metrics (total calcification volume and mean attenuation) in addition to noncalcified components, but it underperformed (ROC‐AUC 0.52), suggesting that aggregation may dilute potentially informative differences between individual calcified foci. Further studies on external datasets are warranted to explicitly study these interactions. Our findings suggest that future streamlined protocols could reasonably focus on a small set of high‐value features—plaque thickness, PVAT, and one or two calcification‐derived metrics—without requiring exhaustive feature extraction. Our CatBoost model's performance aligns with prior evidence that gradient boosting models often outperform other ML approaches, including in stroke prognosis prediction [40, 41].

This study has limitations. It was a single‐center with a modest sample size, resulting in wide confidence intervals and variability. Ulceration, though a recognized high‐risk feature, was rare in our dataset and was not included as a model feature. Manual calcification segmentation introduced variability; no widely available automated tools exist. Because calcification minimum attenuation may be sensitive to noise/partial volume and segmentation boundaries, percentile‐based HU measures should be tested in future validation. Plaque composition relied on commercial software (Elucid Bioimaging), which may limit generalizability. We did not prospectively record manual segmentation or semiautomated segmentation analysis times. The proposed workflow is feasible for research cohorts but would require further streamlining for routine clinical deployment. The recently proposed carotid PLAQUE‐RADS involves IPH volume as part of the criteria; ideally, plaque composition measurements will become more routinely available as software tools mature. An important limitation is that culprit status was defined by laterality. This hemisphere‐based labeling cannot establish that the ipsilateral plaque was truly causal for the embolic event, and contralateral plaques labeled as nonculprit may still share high‐risk features with the ipsilateral plaque. We did not include carotid plaques from stroke‐free individuals, which would represent a more ideal negative reference standard. Our findings should be interpreted in the context of the ESUS definition, which excludes patients with ≥50% ipsilateral stenosis. The performance and feature‐importance patterns we report may not generalize to patients with ≥50% stenosis; the incremental value of PVAT volume, IPH/LRNC ratio, and calcification metrics in that population remain uncertain and will require dedicated non‐ESUS studies. We did not evaluate deep learning models based on raw CTA images; prior convolutional neural network approaches in carotid endarterectomy populations achieved high AUCs [30], but interpretability and applicability to <50% stenotic ESUS populations remain uncertain. We achieved the primary outcome of robust discrimination between culprit and nonculprit plaques with the Combined model (ROC‐AUC 0.79), but probability calibration was a secondary, exploratory analysis and was limited by the modest single‐center test set. In this context, the raw uncalibrated probabilities were overconfident, and standard isotonic post‐hoc calibration improved agreement between predicted and observed risks without significant loss in discrimination. These calibration findings are cohort‐specific, and any future use of model‐derived probabilities for clinical decision‐making would require external validation.

In summary, an explainable classifier using five clinically interpretable CTA‐derived features achieved strong discrimination of culprit plaques in ESUS patients with <50% carotid stenosis and outperformed conventional criteria based on plaque thickness or IPH presence. If validated, this approach could inform imaging‐based risk stratification and guide surveillance or multimodal imaging. SHAP explanations enhance clinical transparency. Through the SHAP framework, the model identified biologically plausible imaging markers, suggested hypothesis‐generating thresholds for future validation, and also allowed visual interpretation of misclassifications. Overall, our results should be viewed as hypothesis‐generating pending external validation. Future directions include longitudinal validation of culprit classifications and comparison with multimodal techniques such as ultrasound and vessel‐wall MRI.

## Funding

YS was supported by NIH/NINIB T‐32 grant (EB004311, PIs Mankoff, Gade, Schnall) and the Foundation of the ASNR 2024 Trainee Research Grant. JWS is funded by the American Heart Association (938082).

## Conflicts of Interest

The authors declare no conflicts of interest.
